# Scalable and cost-effective NGS genotyping in the cloud

**DOI:** 10.1186/s12920-015-0134-9

**Published:** 2015-10-15

**Authors:** Yassine Souilmi, Alex K. Lancaster, Jae-Yoon Jung, Ettore Rizzo, Jared B. Hawkins, Ryan Powles, Saaïd Amzazi, Hassan Ghazal, Peter J. Tonellato, Dennis P. Wall

**Affiliations:** Department of Biomedical Informatics, Harvard Medical School 10 Shattuck Street, Boston, MA 02115 USA; Department of Biology, Mohamed Vth University, 4 Ibn Battouta Avenue, B.P: 1014RP, Rabat, Morocco; Department of Pathology, Beth Israel Deaconess Medical Center, Harvard Medical School, Boston, MA 02215 USA; Department of Electrical, Computer and Biomedical Engineering, University of Pavia, via Ferrata 1, Pavia, 27100 Italy; Department of Biology, Mohamed First University, Oujda, Nador Morocco; Department of Pathology, Brigham and Women’s Hospital, Harvard Medical School, Boston, MA 02215 USA; Department of Pediatrics and Psychiatry (by courtesy), Division of Systems Medicine & Program in Biomedical Informatics, Stanford University, Stanford, CA 94305 USA

**Keywords:** Next-generation sequencing, Clinical sequencing, Cloud computing, Medical genomics, Software, Bioinformatics, Parallel computing

## Abstract

**Background:**

While next-generation sequencing (NGS) costs have plummeted in recent years, cost and complexity of computation remain substantial barriers to the use of NGS in routine clinical care. The clinical potential of NGS will not be realized until robust and routine whole genome sequencing data can be accurately rendered to medically actionable reports within a time window of hours and at scales of economy in the 10’s of dollars.

**Results:**

We take a step towards addressing this challenge, by using COSMOS, a cloud-enabled workflow management system, to develop GenomeKey, an NGS whole genome analysis workflow. COSMOS implements complex workflows making optimal use of high-performance compute clusters. Here we show that the Amazon Web Service (AWS) implementation of GenomeKey via COSMOS provides a fast, scalable, and cost-effective analysis of both public benchmarking and large-scale heterogeneous clinical NGS datasets.

**Conclusions:**

Our systematic benchmarking reveals important new insights and considerations to produce clinical turn-around of whole genome analysis optimization and workflow management including strategic batching of individual genomes and efficient cluster resource configuration.

**Electronic supplementary material:**

The online version of this article (doi:10.1186/s12920-015-0134-9) contains supplementary material, which is available to authorized users.

## Background

Next-generation sequencing costs have plummeted in recent years, rapidly outpacing the traditional benchmark for the decreasing cost of technology known as Moore’s law. Routine clinical whole genome sequencing and analysis now fall within the range of costs of medical testing. Modern sequencing platforms are capable of sequencing approximately 5000 megabases a day [1] at the cost of pennies per megabase. Sequencing centers such as the New York Genome Center, Broad Institute and the Beijing Genomics Institute are now capable of generating petabytes of sequencing data on a routine basis [2]. As a result of the increased efficiency and diminished cost of NGS, the demand for clinical applications is rapidly increasing. Such demand will soon result in large-scale clinical sequence datasets requiring massive data analysis and interpretation at reimbursable cost points thereby producing a technological barrier and price-limiting step of clinical genome usage [3, 4]. Furthermore, achieving practical use of a “clinical” whole genome in routine health care requires a “clinical turnaround” of the sequenced genome rendered to actionable healthcare information at a scale of hours and cost in the $10’s of dollars [4].

With the recent US Food and Drug Administration clearance of Sanger sequencing as a clinical diagnosis tool in January 2013 [5], its subsequent authorization of Illumina deep sequencing technology for similar purposes [6], and the recent announcement by US President Barack Obama of an investment of $200 million in Precision Medicine, cost efficient whole genome sequencing analysis tools and platforms become more critical to the expected implementation across hospitals and clinics. As a result of these recent regulatory developments, delivering a robust software solution that analyzes and renders whole genome NGS into clinically actionable information within hours and under $100 will be a breakthrough in the application of bioinformatics to precision medicine, biomedical science, translational medicine, and healthcare as a whole. In a major step to achieving this goal, we have developed a scalable, parallelizable cloud-enabled workflow management system, COSMOS [7]. COSMOS can optimize whole genome analysis workflows and analysis in two ways: 1) Efficient implementation of highly parallelizable workflows on real or virtual compute clusters, and 2) Options for significant reduction of virtual cluster costs such as use of transient instances invoked and dismissed on-the-fly.

Many NGS processing systems involve the successive implementation of analysis applications into complex environments such as Tavaxy [8], STORMseq [9] and Galaxy [10]. These software packages are generally user-friendly workflow management systems designed for biomedical researchers with relatively little computational experience. More recently, efforts have leveraged the power and speed of highly parallel computing in NGS workflows [11]. As yet, few of these software packages have the speed and throughput necessary for use in large biomedical genomics projects or upcoming projected routine clinical applications, which necessarily involve the cost-effective processing of hundreds of genomes or exomes.

Our past efforts have resulted in significant insight into the technical requirements to leverage the power of cloud computing for NGS [12], and we have extended that approach to developing an NGS analysis workflow, GenomeKey, implemented in COSMOS. GenomeKey performs a thorough sequence analysis, including alignment, quality score recalibration, variant calling and annotation, and can be implemented on either cloud or local high-performance compute clusters. Our selection of tools takes advantage of the runtime performance, cost, and scalability of the GATK best practices standards established by the Broad Institute [13]. GenomeKey’s implementation in COSMOS provides a platform, scalable process and reproducible analyzer of genomic data that can be optimized for speed and cost performance across any cloud or local computing cluster. Herein, we present the results of a comprehensive benchmark study of the COSMOS implementation of GenomeKey on AWS’s cloud services using a heterogeneous combination of public and clinical NGS data to explore speed-to-cost tradeoffs and demonstrate computational barriers requiring further optimization. We show that COSMOS’ execution of GenomeKey reduces the time and cost of whole genome and exome analysis over 10-fold, from published cost estimates of ~ $1000 [4] to under $100 and thus arguably achieves “clinical” turnaround time and reimbursable healthcare costs.

## Methods

**Fig. 1:**
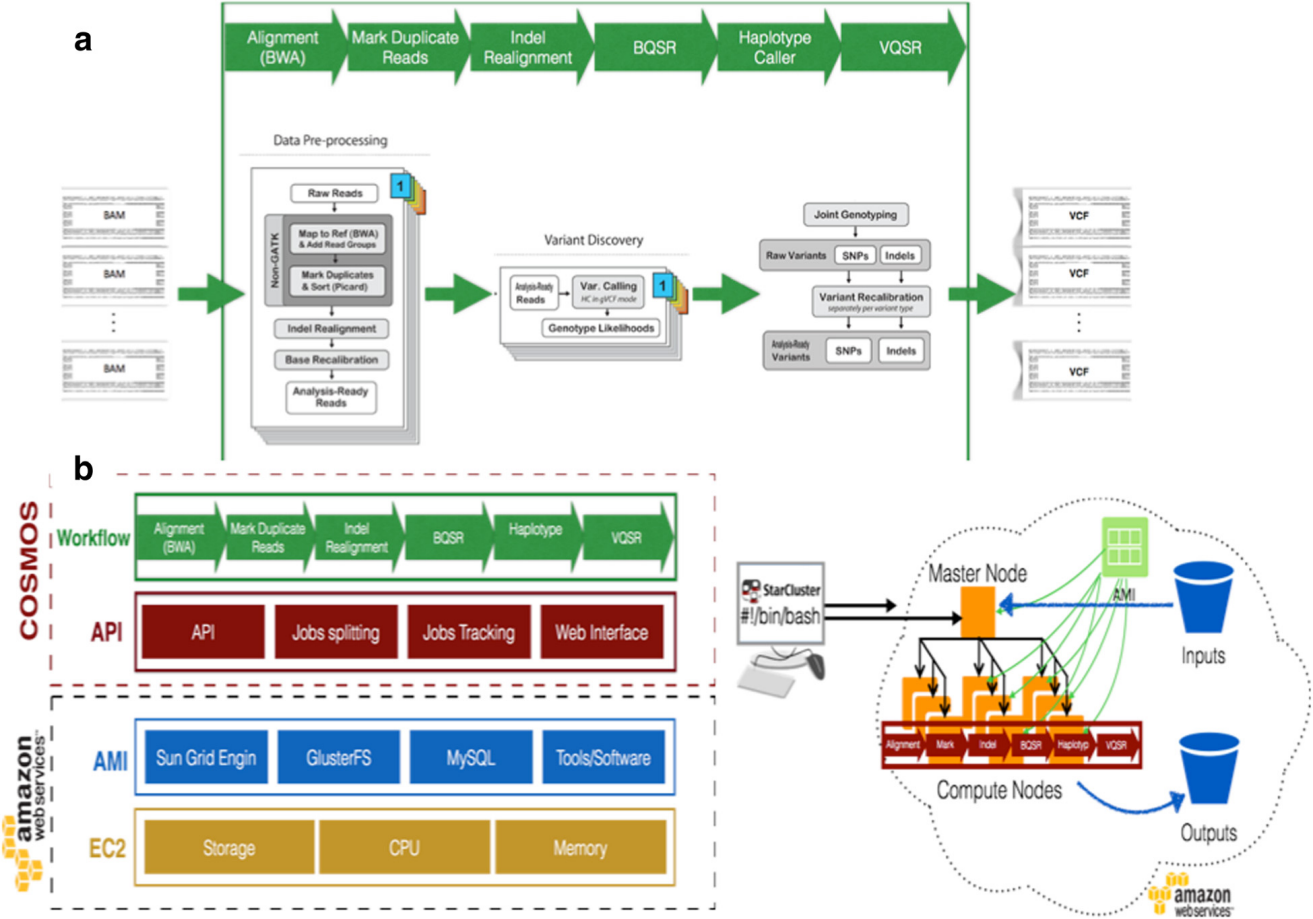
GenomeKey workflow and overall benchmarking study design. **a** GenomeKey workflow implements the GATK 3 best practices for genomic variant calling. Each arrow represents a stage of the workflow, and the level of parallelization for each stage is described in the Methods section under “Workflow”. **b** Deployment of the workflow on the Amazon Web Services Elastic Compute Cloud (EC2) infrastructure using the COSMOS workflow management engine

**Fig. 2:**
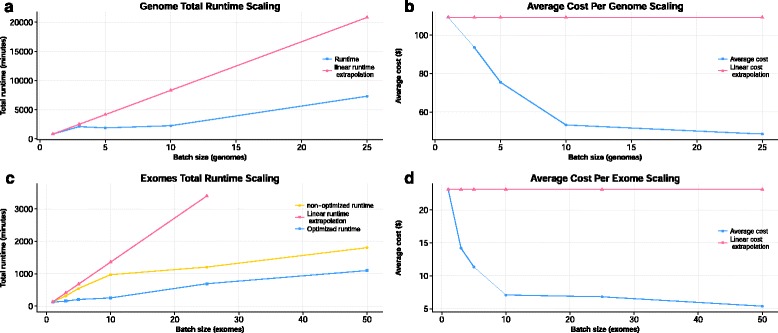
GenomeKey scalability. GenomeKey workflow efficiently scales with increasing number of genomes. **a** Wall time and (**b**) cost as a function of number of genomes compared to a linear extrapolation single genome. GenomeKey workflow scales efficiently with increasing number of exomes compared on different GlusterFS configurations. The blue curve represents the 1, 3, 5 and 10 exomes runs performed on a cluster with one GlusterFS brick; the yellow curve represents the scalability on a cluster with four GlusterFS bricks. **c** Wall time and (**d**) cost as a function of exome and size for as compared to a linear extrapolation of a single exome

**Fig 3:**
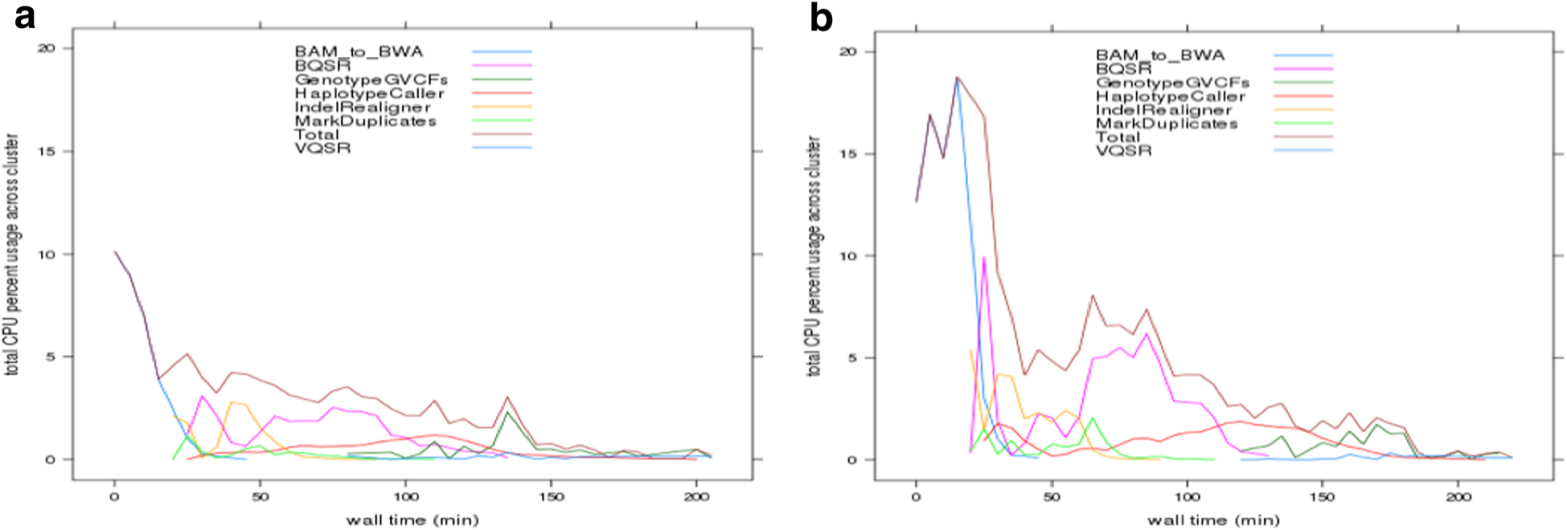
Cluster Resources Usage. Cluster resources are utilized more efficiently as batch size increases. When the number of exomes increases from (**a**) 5 exomes to (**b**) 10 exomes, overall cluster CPU usage (shown as the brown “Total” line) is higher across the entire runtime. Percent CPU usage for each job across the entire 20-node was summed within 5-min “wall time” windows and then scaled by the total number of cores (20 nodes × 32 cores/node = 1920 cores) to quantify the overall system utilization. CPU usage for jobs not fully contained within each 5 min’ window was pro-rated according to how much they overlapped. The contribution of each stage to the entire total (brown line) as a function of time further illustrates the parallelization

Our approach consisted of four methods: (1) COSMOS implementation of GenomeKey workflow to identify genomic variants (Fig. 1a); (2) deployment on Amazon Web Services (AWS) Elastic Compute Cloud (EC2) platform (Fig. 1b); (3) collection of short-read sequencing data for both exomes and genomes; and (4) validation of GenomeKey’s variant calls against published results.

### Workflow

GenomeKey is a Python-based NGS-analysis workflow that implements Genome Analysis Toolkit’s [14] version 3 best practice protocol [13, 15] including alignment, base quality scoring recalibration and joint variant calling for increased statistical power and calibration [14]. GenomeKey is implemented in COSMOS, a Python workflow management system that allows formal description of workflows and partitioning of jobs [7]. GenomeKey’s analysis steps are implemented in COSMOS’s language and tagging system that takes advantage of COSMOS’s parallelization capabilities that supports the map-reduce paradigm [16]. After loading genomic data, GenomeKey “instructs” COSMOS to deconstruct each analysis stage and optimally deploy multiple tasks thereafter managed by COSMOS to run in parallel on available cluster nodes. Although this work was conducted on AWS, the COSMOS/GenomeKey runs equally well on traditional High-Performance Computing clusters.

GenomeKey’s workflow consists of seven stages with an optional annotation stage:

1. Re-alignment and mapping (BAM to BWA). To parallelize realignment: previously aligned BAMs are split into chromosomes, and optionally by read group (RG) using Burrows-Wheeler Aligner [17].
2. Indel realignment (IndelRealign): parallelized by chromosome.
3. Mark read duplicates (MarkDuplicates): parallelized by chromosome.
4. Base quality score recalibration (BQSR): parallelized by sample and chromosome.
5. Generate genomic VCFs (HaplotypeCaller): variants are called per sample and chromosome, generating “gVCF” files: parallelized by sample and chromosome.
6. Genotype samples (GenotypeGVCFs): gVCFs used to call variants jointly across all samples, exomes, or genomes: a serial stage.
7. Variant quality score recalibration (VQSR): parallelized by chromosome for SNPs and Indels separately.
8. Annotation: optional annotation with ANNOVAR databases [18]: parallelized by database chosen for the annotation.

### Deployment on AWS platform

#### Cluster configurations

All runs were performed on a cc2.8xlarge (60 GB of memory, 32 virtual CPUs and 3.3 TB of ephemeral disk) single master node and 20 worker nodes cluster to compare performance and scalability. The master node is an “on-demand” AWS node and has installations of COSMOS and GenomeKey, all tools required for the complete analysis and all input files. Worker nodes can either be “spot-instances”, subject to elimination due to price fluctuation, or fixed price “on-demand” nodes. For “spot-instances”, we placed $0.5/h (~$0.27/h) bids on the us-east AWS region. The ratio of on-demand to spot-instances nodes varied depending on overall size of the datasets. This approach allowed, incorporation of the on demand worker node’s hard drive space into a common pool using GlusterFS (Table 1) in three different configurations:

**Table 1:** GlusterFS configurations used to increase shared disk space

	GlusterFS bricks	Shared Disk Size (TB)
Config 1 (1;0;20)	1	3.3
Config 2 (1;1;19)	2	6.6
Config 3 (1;3;16)	4	13.2

*(L; M; N): L* on-demand master nodes, *M* on-demand worker nodes, *N* spot-instance worker nodes

**Table 2:** Comparison of variant calls results

Variant calls	Ti/Tv All SNPs	High quality SNPs	Genotype concordance
GenomeKey	2.25	202290	0.97
DePristo	2.26	141618	-

#### Cluster management system

The cluster launching and management was performed by the open source software StarCluster (v 0.95.4 on public github repository; http://web.mit.edu/stardev/cluster/). Instances were launched with StarCluster using the Ubuntu 12.04LTS based AMI (Amazon Machine Image) ami-5bd1c832 and by default loaded with COSMOS, GenomeKey as well as the tools needed by GenomeKey: GATK, SAMtools [19].

#### Job management and shared file system

Jobs created by COSMOS are submitted and managed across nodes in the cluster using Sun Grid Engine 6.2u5-4. The compute nodes of the cluster share a common scratch space provided by one or more node(s). This shared filesystem is created and managed by GlusterFS 3.4 (https://www.gluster.org), a cluster shared file system.

#### Computing AWS cost

We used the AWS cli tools (http://aws.amazon.com/cli/) to compute the cost of “spot” instances using the start and stop timestamps of the workflow recorded by GenomeKey and our study driver automation script (Additional file 1: Figure S2), specifically using the following command script:

aws ec2 describe-spot-price-history --start-time [start timestamp] --end-time [end timestamp] --instance-types cc2.8xlarge --availability-zone

The command returns the history of the spot price during the specified period and the mean of the returned values is used to compute spot instance cost. On-demand prices are fixed by AWS (https://aws.amazon.com/ec2/pricing). Total cluster cost was computed by adding on-demand and spot node costs.

### Benchmark data

Benchmark run times and costs were computed for an increasing number of NGS datasets run on the fixed 21 node cluster by defining a range of groups of exomes and genomes of size, n. For exomes, *n* = {1, 3, 5, 10, 25, 50}. For genomes *n* = {1, 5, 10, 25}.

#### Exomes

We used high coverage (~150x) whole exomes of the CEU trio (NA12878, NA12891 and NA12892; Additional file 2: Table S1) from the Coriell CEPH/UTAH 1463 pedigree, sequenced at Broad Institute and recommended by GATK for review or benchmark purposes (http://gatkforums.broadinstitute.org/discussion/1292/which-datasets-should-i-use-for-reviewing-or-benchmarking-purposes.). These individuals also have high coverage whole genome datasets and have extensive published results. We included this trio in each exome run, in order to compare and crosscheck the quality of output variants (of the same input data) over different runs (Additional file 2: Table S1).

To round out the full exome dataset panel, we included biomedically disease relevant exomes originally generated by Christopher Walsh’s group [20]. These samples were chosen because (i) the data is curated National Database for Autism Research (NDAR); ii) mean read depth for proband data (~158x) matches up with that of CEU trio; iii) extended family data are available including affected siblings; iv) VCF files are provided for a subset of probands; and v) phenotype information is available via NDAR or AGRE. The BAM files are renamed to group by families. This dataset was also sequenced at Broad Institute, so we used the exome target regions (Agilent Sure-Select Human All Exon v2.0, 44 Mb baited target) provided in the GATK bundle, with 100 bp padded at both ends, to extract targets for both control/case exome data.

#### Genomes

##### BGI genomes

We selected 31 unique autism-associated genomes with coverage ranging from 31.5x to 42x with a mean coverage of 37x originally sequenced by BGI on Illumina platform. The genomes were selected to have trios in each run to take advantage of the joint variants calling feature of GenomeKey. Pedigree information for these genomes is also available (Additional file 2: Table S1).

##### Platinum genomes

We selected a single high coverage genome (~50x), sample NA12878 (one of the Exome trio described in 2.3.1). The Platinum genomes also have “gold standard” variant calls in VCF format with variants called using different software and technology. Those VCFs enable quality control (see below), as well as reference timings from Blue Collar Bioinformatics group bcbio-nextgen. (http://www.illumina.com/platinumgenomes/).

#### Variant validation

To validate GenomeKey, we downloaded previously generated BAM files available for the trio of exomes from Phase I of the 1000 Genomes Project. For the 1000 Genomes trio, we were then able to compare our BAMs with these downloaded BAMs and quality control included:

1. Variant quality score recalibration (VQSR) Compared the percentage of unmapped reads between our original mapped BAM and our re-mapped BAM. Although the number of mapped reads may be different to the Phase I output because of BWA and reference genome version differences, we anticipate very similar mapped reads.
2. Compared the distribution of phred base quality scores for each paired BAM files using FastQC.

We compared the results of GenomeKey variant calls against available benchmark data [15]. The analysis was performed on NA12878 whose corresponding exome BAM file was originally aligned with MAQ on hg18. In order to compare variant calls we ran the same method (GATK v3 HaplotypeCaller) over the benchmark’s BAM and our re-mapped BAM. The procedure was set with identical parameters though different reference genome. An additional analysis was performed on sequencing data publicly available at www.platinumgenomes.org. The platinum genomes are whole genome sequence and variant call data for 17 members of the Coriell CEPH/UTAH, including NA12878, in order to create a “platinum” comprehensive set of variant calls. We extracted raw sequence data from NA12878’s BAM files and re-mapped them against hg19 with GenomeKey. Resulting VCFs were tested for concordance against benchmark whole-genome VCFs from the Genome in a Bottle Consortium [21].

## Results

GenomeKey implemented in COSMOS was deployed on AWS cloud cluster (Table 1) to process successively larger sets of whole exome (1,3,10, 25, 50) and whole genome (1, 3, 10, 25) datasets. Exomes and Genomes were split and parallelized by chromosome and read-groups. Both overall wall time and timepoints for each analysis stage were benchmarked. Costs were calculated using standard AWS costs.

### Scalability and robust handling of heterogeneous datasets

A heterogeneous collection of exomes and genomes were selected to test the robustness and scalability of the system. The data included genomes from the 1000 Genomes Project, autism exomes, autism genomes, and Illumina’s “Platinum” genomes (Additional file 2: Table S1) (http://www.illumina.com/platinumgenomes/.) This diverse collection enabled assessment of the performance parameters of the workflow with relatively homogeneous versus heterogeneous data, as expected in both biomedical and clinical scenarios. Exomes and genomes were batched (1, 3, 5, 10, 25 and 50 for exomes and 1,3,5,10,25 for genomes) to test scalability. COSMOS managed the processing of both heterogeneous genomic data inputs as well as increasing numbers of genomic data across the entire panel of testing with only two disrupted runs (*n* = 25 genomes.) Each disruption was the result of the loss of a single “spot” AWS worker node that COSMOS rescued with its recovery and resume function capabilities, enabling a clean restart at the check-point just before the disruption to ensure successful completion of the run with little additional cost.

### Accuracy

GenomeKey alignment accuracy was tested by a trio of exomes from Phase I of the 1000 Genomes Project [22], which were remapped and tested against the original mapping. Various differences between original mapping and that performed in this study can cause differences in the mapped reads (e.g. different versions of analysis tools such as BWA). Our analysis demonstrated a ~0.25 % increase in total mapped reads over the original published results. FastQC was used to compare the phred base quality scores over the reads. The original BAMs were re-calibrated with GATK and overall quality of sequence length was higher for the BAM files generated by GenomeKey (Additional file 3: Table S2).

GenomeKey variant call accuracy was tested against available benchmark data [15]. In particular, one genome (NA12878) was used to generate a collection of quality variant metrics (see Methods and Additional file 4: Table S3 for details). Genotype concordance as reported by the GATK Genotype Concordance module was 0.97 (Table 2).

Genotype concordance was also compared against the Genome in the Bottle Consortium (www.genomeinabottle.org) benchmark whole-genome genotype calls dataset designed to minimizes bias from any one method by integrating 14 datasets from five sequencing technologies, seven read mappers and three variant callers [21]. GenomeKey’s analysis of the genome resulted in an overall genotype concordance (3,055,906 matching alleles and 7368 non-matching alleles) and 0.997 sensitivity with the Zook et al. results.

### Baseline runtimes for collections of exomes and genomes

COSMOS’s execution of GenomeKey on small collections of high-coverage genomes provided baseline runtime speeds and costs (Fig. 2). A high-coverage (~150×) exome from alignment to variant calling runtime was 136 min for a total cost of $23 (download and backup to AWS S3 storage cost $5). Four replicate runs of 1, 3, 5 and 10 exome batched analysis (using similarly characterized genomes), established robust runtime and cost estimates with low variance. (e.g. 1 exome analysis runtime had a mean of 123 +/− 2.3 min) (Fig. 2c and Additional file 5: Figure S1). A single genome (42× coverage) analysis was 13 h 52 mins for a total cost of ~ $109 (Fig. 2a and b). The cost compares favorably with other tools (see Discussion). COSMOS’ ability to use AWS Spot Instances reduces the overall costs from $588 to $109 in the single whole genome case.

Similarly, to the 1 exome run, a 3 exomes runtime had a mean of 158 min +/− 366 s and an average cost of $14.19, the 5 exomes runtime had a mean of 206 +/− 719 s and a cost of $11.35, the 10 exomes runtime had a mean of 255 min +/− 1673 s, the 25 exomes and 50 exomes run were not systematically replicated and the total runtimes are respectively 11 h 32 min and 18 h 19 min, and costs are respectively $170 and $270. The 1 genome run was 13 h 53 min and cost under $110, 3 genomes run in 35 h 10 min and cost $280, 5 genomes run in 31 h and 31 min and cost $377, 10 genomes run in 37 h and 48 min and cost $533 and the 25 genomes run in 121 h and cost $1213 (Additional file 6: Table S4, Fig. 2a and b).

### COSMOS parallelization

Strategically batched exome and genome datasets using COSMOS’ parallelization features result in significant savings in runtime and costs especially when testing large runs (50 and above). In particular, GenomeKey’s alignment steps (consistent with most whole genome analysis workflow alignment steps) demonstrate a sub-linear scaling for both genomes (Fig. 2a) and exomes (Fig. 2c). These efficiencies are tied to the ability of COSMOS to optimize the recruitment and use of available CPU cycles and worker nodes thus using the cluster resource closer to maximum capacity across the entire GenomeKey workflow and corresponding analysis steps (Fig. 3). The design of this Baseline study matching scalability with fixed 21-node cluster was created to test the limits of scalability of a fixed cluster resource. In this case, the 21-node cluster reached computational capacity and, therefore, displayed relatively increased runtimes and costs at 50 exomes. Batching multiple samples reduces the per-sample analysis cost and average runtime. However the batching strategy results in a longer time-to-completion per sample. This is due to the fact that while alignment itself can be parallelized, all alignments for a given chromosome must be completed before joint variant-calling can be performed, effectively making the final step a single serial step resulting in each NGS sample completed and ready at the total wall time of the entire workflow. As an example, a single exome processing time is ~2 h compared to ~12 h for 25 batched exomes (a 10 h ‘delay’ compared to a single exome run). However, this modest delay results in a dramatic four-fold reduction in the cost per exome, $5.81 compared to $25.37 (Fig. 2b, d and Additional file 6: Table S4).

### COSMOS storage management

Several modes of storage management were tested to improve COSMOS’ GenomeKey stage-to-stage data sharing across multiple worker nodes. We increased the number of nodes participating in the GlusterFS shared volume from 2 to 4 instances, and the performance was measured using the same input data. This reduced the runtime two fold in the 25 exome run from ~20 h to ~11.5 h (Fig. 3a). The per-stage runtime gain was measured, and the data transfer for the alignment stage (BWA) was found 2.5 times faster using the 4-node GlusterFS configuration compared with the 2-node configuration (Fig. 3).

### COSMOS next-generation parallelization

We measured the impact of two parallelization strategies of the alignment stage of GenomeKey (“BAM to BWA”). The first is splitting the input bam BAM files by chromosomes as well as read-group (corresponding to an individual “lane” for Illumina data), and the second is to split with chromosome only (an option of GenomeKey). The impact of the extra parallelization in the first strategy offers a modest reduction in runtime compared to the second strategy for a single exome. However, for a larger batch size (25 'omes and higher), the number of generated tasks overwhelms the queuing system.

## Discussion

Our results demonstrate that the COSMOS implementation of GenomeKey provides a scalable, robust and efficient solution to address the ever-increasing demand for efficient, cost-effective genomic sequence analysis. COSMOS’ workflow management features offer parallelization and storage options that dramatically improve overall performance and reduce costs of whole genome and exome analysis pipelines with the greatest improvements in the computationally intensive alignment stage. In addition, other analysis stages (i.e. variant calling), runtime and cost improvements reflect strategically batched NGS datasets (e.g. by chromosome). Our runtime and cost benchmarking verified robustness and scalability of COSMOS’ implementation of GenomeKey for large collections of exomes and genomes when run on fixed-resourced cloud clusters. These results also for the first time establish standardized runtimes, resource use and demonstrate scalability parameters and per-exome and genome costs and provides valuable qualitative comparisons with existing approaches and methods. Our systematic approach also yielded actionable guidance for researchers using COSMOS, GenomeKey (or potentially other GATK-based workflows) by quantifying configuration choices in two key areas: speed-cost tradeoffs in the number of exomes or genomes processed and choice and configuration of cloud cluster and filesystem. We discuss each of these below.

### Run-time and cost comparisons with other cloud-based genome analysis workflows

User-friendly sequence analysis systems (e.g. Galaxy) have demonstrated flexibility, user-friendly interface and robust workflow design especially for users without deep bioinformatics experience. COSMOS and GenomeKey, with less user-friendly features, provide important new options and configurations that provide efficient, low-cost, and robustly accurate sequence analysis. Community standards for performance benchmarking of exomic and genomic analysis workflows are not established, so few results are published and comparisons (either qualitative or quantitative) between software packages are generally unavailable. Rather, feature chart comparisons as seen in SIMPLEX [23], or general qualitative comparisons, as seen in Mercury [24] are available. Runtime characteristics for individual systems are available, however, those analyses are not easily comparable across systems, cluster configurations or by grouped ‘platinum’ datasets thus few options exist to identify specific barriers and computational inefficiencies between workflows. Individual publications of each workflow typically report runtime statistics on a variety of different datasets, hardware configuration, and storage options (Additional file 6: Table S4 and Additional file 7: Table S5).

COSMOS’s implementation of GenomeKey offers a fast, cost-efficient, accurate solution for alignment, cleaning, and variant-calling of genomes and exomes with better performance compared to other software. STORMseq [9], uses BWA for alignment and GATK lite for quality control and variant calling, processed an exome in 10 h and a genome in 176 h, compared to GenomeKey which completed an exome and a genome run in 2 h and 14 h, respectively. Other software packages have been found to perform at similar levels to STORMseq, with exomes being processed in 10–25 h (see 7: Table S5 for more details). However, these results are not direct comparisons since different datasets and different computational resources were used for each software package. Additionally, many of these systems prioritize ease of use on small-scale datasets [10] rather than focusing on processing large-scale datasets typical of biomedical research and projected clinical needs. One large-scale benchmarking effort has been published using the cloud-based, genomics workflow, Rainbow [25] which processed 44 genomes in two weeks for $5800, ~$120 per genome. COSMOS’s GenomeKey compares favorably at $50 per genome for up to 25 genomes. If two 21-node clusters were deployed, and the Rainbow dataset broken into 2 sets of 22 genomes each, we extrapolate that the use of GenomeKey would result in a $53.36 per genome cost, processed 1088 low-coverage genomes (around 62 million reads) in under 7 days (168 h) using 400 AWS CC2.8xlarge nodes. These low-coverage genomes have on average 62 million reads this is equivalent to 8 whole genomes at 36× coverage (500 million reads). Using our optimal batching strategy, with 14 21-node clusters were to be deployed, and the 1088 data broken into 13 sets of 80 genomes and one set of 48 genomes, we extrapolate that GenomeKey would accomplish the analysis in 32 h with and estimated cost of $7500 compared to an estimated $16,600 for Churchill [26] (assuming all the 400 spot instances).

### A road-map to routine, cost effective, clinical-turnaround, whole genome analyses

The systematic benchmarking of COSMOS’ GenomeKey allowed us to build and investigate a “complexity roadmap” of NGS variant calling workflows in the cloud. Particularly, the results provide guidance in two keys areas: (1) choosing the optimum batch size and (2) choosing optimum shared storage configurations.

In a clinical setup, the price point for genomic analysis per-sample is fixed. Using the collected metrics of cost and runtime per exome and genome, we can provide batch size estimates to achieve a time-to-sample analysis completion under a day and for a minimal cost. This also depends on the nature of the generated sequencing data. In the case of the autism exomes, there were upwards of 10 read-groups per sample, but it clearly illustrates that there is an upper limit to parallelization of the workflow (alignment stage). We thus provide a command-line option to the GenomeKey workflow to split by chromosome only instead of the default chromosome and read-group.

Using GlusterFS file-system (see Methods) to pool hard-drive storage across multiple nodes dramatically improved overall runtime. Even the fact that GenomeKey is setup to limit the transfer from and to the shared file system within running stages, this limits network latency. However, the output of each stage needs to be saved on shared storage for dependent tasks to proceed to the next stage and backup in the case of spot instance or other node failures. Our benchmarking reveals that shared storage use requires significant time and proves the value of a multiple shared storage node configuration. Shared storage optimization becomes particularly important for large batch sizes, with many jobs performing parallelized intensive reads and writes. The shared nodes in the GlusterFS configuration must be persistent (on-demand) AWS instances and thus are more expensive than transient (spot) instances. However, this tradeoff was more than offset by overall processing speed improvement. In our particular case, a batch of 10 to 15 exomes offers an excellent time and cost balance. This heuristic might vary depending on the sequencing analysis and computational insight of a given COSMOS-GenomeKey user.

### Availability and implementation

COSMOS code is available for academic non-commercial research purposes; GenomeKey is available under an MIT open source license. The source code of COSMOS and GenomeKey as well as the documentation and support information are available on the project website at http://cosmos.hms.harvard.edu and via GitHub repository at https://github.com/LPM-HMS/COSMOS and https://github.com/LPM-HMS/GenomeKey, respectively.

## Additional files

Additional file 1: Figure S2. Automation script. Organogram of the automation script that downloads the data, run the pipeline, save all the steps timestamps and backup the data. (PNG 64 kb) Additional file 2: Table S1. Input datasets per run. (PDF 215 kb) Additional file 3: Table S2. FastQC quality control table. (PDF 302 kb) Additional file 4: Table S3. Comparison with existing implementations of the GATK best practices, features. (PDF 19 kb) Additional file 5: Figure S1. Exomes total runtime scaling. Replicated exomes runs, shows highly reproducible runtimes. Error bars represent variance. (PNG 43 kb) Additional file 6: Table S4. Runs summary with detailed cluster configuration, mean size, coverage, splitting strategy (chr: chromosome, RG: read-group), total runtime, total cost, average runtime and average cost. (PDF 61 kb) Additional file 7: Table S5 Comparison with previous benchmarks for specific 1000 g exomes/genomes. (PDF 26 kb)

## Abbreviations

AWS: Amazon Web Services
BAM: Binary Alignment/Mapping
BQSR: Base quality score recalibration
BWA: Burrows-Wheeler Aligner
CPU: Central processing unit
GATK: Genome analysis tool kit
hg18/19: Human genome build 18/19
Indel: Insertion/deletion
MAQ: Mapping and Assembly with Qualities
NGS: Next-generation sequencing
S3: Simple storage service
SNP: Single nucleotide polymorphism
VCF: Variant call format
VQSR: Variant quality score recalibration

## References

[1] Kircher M, Kelso J (2010). High-throughput DNA sequencing--concepts and limitations. Bioessays.

[2] Schatz MC, Langmead B (2013). The DNA data deluge: fast, efficient genome sequencing machines are spewing out more data than geneticists can analyze. IEEE Spectr.

[3] Desai AN, Jere A (2012). Next-generation sequencing: ready for the clinics?. Clin Genet.

[4] Sboner A, Mu XJ, Greenbaum D, Auerbach RK, Gerstein MB (2011). The real cost of sequencing: higher than you think!. Genome Biol.

[5] Life Technologies Receives FDA 510(k) Clearance for Diagnostic Use of Sanger Sequencing Platform and HLA Typing Kits [https://www.genomeweb.com/sequencing/510k-clearance-3500-dx-life-tech-aims-convert-hla-typing-customers-cleared-box-a]

[6] Collins FS, Hamburg MA (2013). First FDA authorization for next-generation sequencer. N Engl J Med.

[7] Gafni E, Luquette LJ, Lancaster AK, Hawkins JB, Jung JY, Souilmi Y (2014). COSMOS: python library for massively parallel workflows. Bioinformatics.

[8] Abouelhoda M, Issa SA, Ghanem M (2012). Tavaxy: integrating Taverna and Galaxy workflows with cloud computing support. BMC Bioinformatics.

[9] Karczewski KJ, Fernald GH, Martin AR, Snyder M, Tatonetti NP, Dudley JT (2014). STORMSeq: an open-source, user-friendly pipeline for processing personal genomics data in the cloud. PLoS One.

[10] Goecks J, Nekrutenko A, Taylor J, Galaxy T (2010). Galaxy: a comprehensive approach for supporting accessible, reproducible, and transparent computational research in the life sciences. Genome Biol.

[11] Nekrutenko A, Taylor J (2012). Next-generation sequencing data interpretation: enhancing reproducibility and accessibility. Nat Rev Genet.

[12] Fusaro VA, Patil P, Gafni E, Wall DP, Tonellato PJ (2011). Biomedical cloud computing with Amazon Web Services. PLoS Comput Biol.

[13] Van der Auwera GA, Carneiro MO, Hartl C, Poplin R, Del Angel G, Levy-Moonshine A (2013). From FastQ data to high confidence variant calls: the Genome Analysis Toolkit best practices pipeline. Curr Protoc Bioinformatics.

[14] McKenna A, Hanna M, Banks E, Sivachenko A, Cibulskis K, Kernytsky A (2010). The Genome Analysis Toolkit: a MapReduce framework for analyzing next-generation DNA sequencing data. Genome Res.

[15] DePristo MA, Banks E, Poplin R, Garimella KV, Maguire JR, Hartl C (2011). A framework for variation discovery and genotyping using next-generation DNA sequencing data. Nat Genet.

[16] Dean J, Ghemawat S (2008). MapReduce: simplified data processing on large clusters. Commun ACM.

[17] Li H, Durbin R (2009). Fast and accurate short read alignment with Burrows-Wheeler transform. Bioinformatics.

[18] Wang K, Li M, Hakonarson H (2010). ANNOVAR: functional annotation of genetic variants from high-throughput sequencing data. Nucleic Acids Res.

[19] Li H, Handsaker B, Wysoker A, Fennell T, Ruan J, Homer N (2009). The sequence alignment/Map format and SAMtools. Bioinformatics.

[20] Yu TW, Chahrour MH, Coulter ME, Jiralerspong S, Okamura-Ikeda K, Ataman B (2013). Using whole-exome sequencing to identify inherited causes of autism. Neuron.

[21] Zook JM, Chapman B, Wang J, Mittelman D, Hofmann O, Hide W (2014). Integrating human sequence data sets provides a resource of benchmark SNP and indel genotype calls. Nat Biotechnol.

[22] Abecasis GR, Altshuler D, Auton A, Brooks LD, Durbin RM, Genomes Project C (2010). A map of human genome variation from population-scale sequencing. Nature.

[23] Fischer M, Snajder R, Pabinger S, Dander A, Schossig A, Zschocke J (2012). SIMPLEX: cloud-enabled pipeline for the comprehensive analysis of exome sequencing data. PLoS One.

[24] Reid JG, Carroll A, Veeraraghavan N, Dahdouli M, Sundquist A, English A (2014). Launching genomics into the cloud: deployment of Mercury, a next generation sequence analysis pipeline. BMC Bioinformatics.

[25] Zhao S, Prenger K, Smith L, Messina T, Fan H, Jaeger E (2013). Rainbow: a tool for large-scale whole-genome sequencing data analysis using cloud computing. BMC Genomics.

[26] Kelly BJ, Fitch JR, Hu Y, Corsmeier DJ, Zhong H, Wetzel AN (2015). Churchill: an ultra-fast, deterministic, highly scalable and balanced parallelization strategy for the discovery of human genetic variation in clinical and population-scale genomics. Genome Biol.

