# Supplementary figures and images for: Scalable and cost-effective NGS genotyping in the cloud
Source: BMC Med Genomics. 2015 Oct 15;8:64. doi: 10.1186/s12920-015-0134-9 (PMC4608296; doi:10.1186/s12920-015-0134-9)

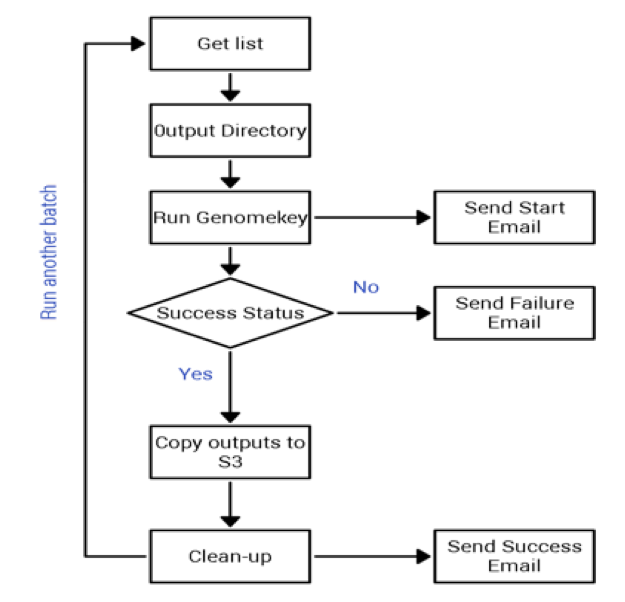

Supplement: Additional file 1: Figure S2. — Automation script. Organogram of the automation script that downloads the data, run the pipeline, save all the steps timestamps and backup the data. (PNG 64 kb) [file 12920_2015_134_MOESM1_ESM.png]

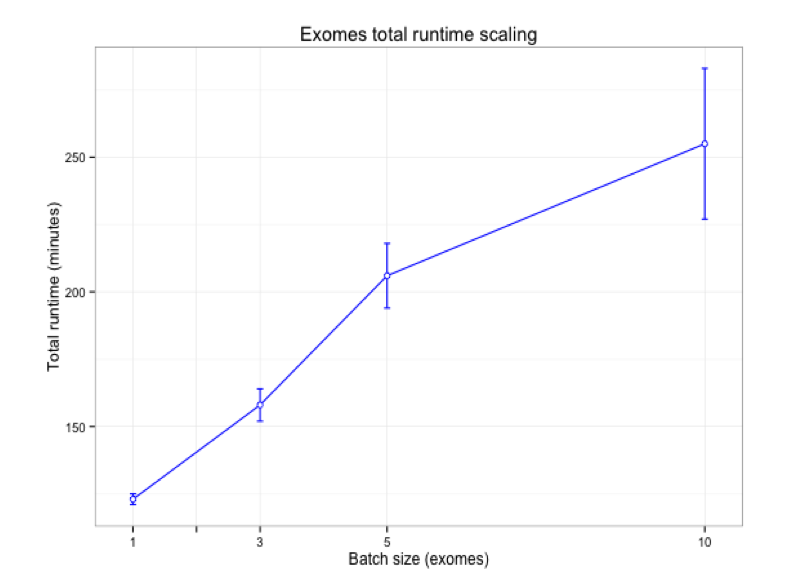

Supplement: Additional file 5: Figure S1. — Exomes total runtime scaling. Replicated exomes runs, shows highly reproducible runtimes. Error bars represent variance. (PNG 43 kb) [file 12920_2015_134_MOESM5_ESM.png]
