# Supplementary material for: Scalable and cost-effective NGS genotyping in the cloud
Source: BMC Med Genomics. 2015 Oct 15;8:64. doi: 10.1186/s12920-015-0134-9 (PMC4608296; doi:10.1186/s12920-015-0134-9)
Supplement: Additional file 2: Table S1. — Input datasets per run. (PDF 215 kb) [file 12920_2015_134_MOESM2_ESM.pdf]

Table S1 Input datasets per run

Run	Sample	Reads length	Coverage	Size (GB)	Family ID	Paternal ID	Maternal ID	Sex	Phenotype	Mean size (GB)	Mean Coverag
**1 EX**	NA12878	76bp	150x	26.45	CEPH 1463			F	Control	**26.45**	**150x**
**3 EX**	NA12878	76bp	150x	26.45	CEPH 1463			F	Control	**27.27**	**150x**
	NA12891	76bp	150x	25.85	CEPH 1463			M	Control		
	NA12892	76bp	150x	29.52	CEPH 1463			F	Control		
**5 EX**	NA12878	76bp	150x	26.45	CEPH 1463			F	Control	**24.68**	**162x**
	NA12891	76bp	150x	25.85	CEPH 1463			M	Control		
	NA12892	76bp	150x	29.52	CEPH 1463			F	Control		
	AU-0352a	50bp	206x	23.72		0	0	M	Autism		
	AU-0625a	50bp	155x	17.87		0	0	F	Autism		
**10 EX**	NA12878	76bp	150x	26.45	CEPH 1463			F	Control	**18.75**	**137x**
	NA12891	76bp	150x	25.85	CEPH 1463			M	Control		
	NA12892	76bp	150x	29.52	CEPH 1463			F	Control		
	AU-0812a	50bp	178x	20.45		0	0	F	Autism		
	AU-0753a	50bp	160x	18.35		0	0	M	Autism		
	AU-11901	50bp	185x	21.24	AU-11900	AU-11905	AU-11904	F	Autism		
	AU-11902	50bp	148x	16.97	AU-11900	AU-11905	AU-11904	M	Autism		
	AU-11903	50bp	139x	15.98	AU-11900	AU-11905	AU-11904	M	Autism		
	AU-11904	50bp	56x	6.38	AU-11900	0	0	F	Control		
	AU-11905	50bp	54x	6.26	AU-11900	0	0	M	Control		
**25 EX**	NA12878	76bp	150x	26.45	CEPH 1463			F	Control	**17.83**	**145x**
	NA12891	76bp	150x	25.85	CEPH 1463			M	Control		
	NA12892	76bp	150x	29.52	CEPH 1463			F	Control		
	AU-1196a	50bp	194x	22.3		0	0	M	Autism		
	AU-1019a	50bp	160x	18.35		0	0	M	Autism		

	AU-1210	50bp	145x	16.71		0	0	M	Autism		
	AU-1206	50bp	211x	24.31	AU-1200	AU-1210		M	Autism		
	AU-1203	50bp	102x	11.73	AU-1200	AU-1210		F	Control		
	AU-1201	50bp	188x	21.57	AU-1200	AU-1210		F	PDD-NOS		
	AU-15504	50bp	86x	9.94	AU-15500	0	0	M	Control		
	AU-15503	50bp	82x	9.45	AU-15500	0	0	F	Control		
	AU-15502	50bp	215x	24.72	AU-15500	AU-15504	AU-15503	M	Autism		
	AU-15501	50bp	224x	25.78	AU-15500	AU-15504	AU-15503	M	Autism		
	AU-1804	50bp	87x	9.98	AU-1800	0	0	M	Control		
	AU-1803	50bp	86x	9.88	AU-1800	0	0	F	Control		
	AU-1802	50bp	224x	25.77	AU-1800	AU-1803	AU-1804	F	Autism		
	AU-1801	50bp	209x	24.07	AU-1800	AU-1803	AU-1804	M	Autism		
	AU-1707	50bp	66x	7.55	AU-1700	AU-1704	AU-1703	M	Control		
	AU-1704	50bp	80x	9.2	AU-1700	0	0	M	Control		
	AU-1703	50bp	85x	9.74	AU-1700	0	0	F	Control		
	AU-1702	50bp	218x	25.03	AU-1700	AU-1704	AU-1703	M	Autism		
	AU-1904	50bp	82x	9.48	AU-1900	0	0	F	Control		
	AU-1903	50bp	145x	16.66	AU-1900	NDAR_INVPE918ER1	AU-1904	M	Autism		
	AU-1902	50bp	144x	16.51	AU-1900	NDAR_INVPE918ER1	AU-1904	M	Autism		
	AU-1901	50bp	132x	15.22	AU-1900	NDAR_INVPE918ER1	AU-1904	M	Autism		
**50 EX**	NA12878	76bp	150x	26.45	CEPH 1463			F	Control	**17.01**	**143x**
	NA12891	76bp	150x	25.85	CEPH 1463			M	Control		
	NA12892	76bp	150x	29.52	CEPH 1463			F	Control		
	AU-1462a	50bp	162x	18.61		0	0	F	Autism		
	AU-1388b	50bp	189x	21.72		0	0	M	Autism		
	AU-16701	50bp	217x	24.99	AU-16700	AU-16703	AU-16702	M	Autism		
	AU-16702	50bp	89x	10.28	AU-16700	0	0	F	Control		
	AU-16703	50bp	179x	20.52	AU-16700	0	0	M	Autism		
	AU-2201	50bp	222x	25.51	AU-2200	AU-2205	AU-2204	M	Autism		
	AU-2204	50bp	84x	9.66	AU-2200	0	0	F	Control		

	AU-2205	50bp	87x	10.00	AU-2200	0	0	M	Control		
	AU-2207	50bp	201x	23.16	AU-2200	AU-2205	AU-2204	M	Autism		
	AU-23101	50bp	202x	23.24	AU-23100		NDAR_INVAC924 LHZ	F	Autism		
	AU-23102	50bp	200x	23.04	AU-23100		NDAR_INVAC924 LHZ	F	Autism		
	AU-23103	50bp	56x	6.45	AU-23100		NDAR_INVAC924 LHZ	M	Control		
	AU-23801	50bp	182x	20.93	AU-23800	AU-23803	AU-23802	M	Autism		
	AU-23802	50bp	135x	15.56	AU-23800	0	0	F	Control		
	AU-23803	50bp	116x	13.33	AU-23800	0	0	M	Control		
	AU-2401	50bp	218x	25.06	AU-2400	AU-2405	AU-2404	M	Autism		
	AU-2404	50bp	56x	6.42	AU-2400	0	0	F	Control		
	AU-2405	50bp	55x	6.33	AU-2400	0	0	M	Control		
	AU-25901	50bp	210x	24.13	AU-25900	AU-25904	AU-25903	F	Autism		
	AU-25902	50bp	71x	8.16	AU-25900	AU-25904	AU-25903	F	Autism		
	AU-25903	50bp	55x	6.35	AU-25900	0	0	F	Control		
	AU-25904	50bp	51x	5.86	AU-25900	0	0	M	Control		
	AU-26001	50bp	158x	18.16	AU-26000	AU-26006	AU-26005	M	Autism		
	AU-26005	50bp	107x	12.30	AU-26000	0	0	F	Control		
	AU-26006	50bp	177x	20.36	AU-26000	0	0	M	Control		
	AU-26501	50bp	177x	20.39	AU-26500	AU-26504	AU-26503	F	Autism		
	AU-26503	50bp	221x	25.42	AU-26500	0	0	F	Control		
	AU-26504	50bp	150x	17.21	AU-26500	0	0	M	Control		
	AU-26601	50bp	186x	21.43	AU-26600	AU-26604	AU-26603	M	Autism		
	AU-26603	50bp	113x	12.97	AU-26600	0	0	F	Control		
	AU-26604	50bp	153x	17.58	AU-26600	0	0	M	Control		
	AU-2901	50bp	220x	25.34	AU-2900	AU-2908	AU-2907	M	Pervasive Developmental Disorder Not Otherwise Specified		
	AU-2902	50bp	213x	24.43	AU-2900	AU-2908	AU-2907	M	Autism		
	AU-2907	50bp	56x	6.41	AU-2900	0	0	F	Control		
	AU-2908	50bp	115x	13.20	AU-2900	0	0	M	Control		
	AU-3001	50bp	51x	5.83	AU-3000	AU-3005	AU-3004	F	Autism		
	AU-3003	50bp	386x	44.35	AU-3000	AU-3005	AU-3004	F	Control		

	AU-3004	50bp	55x	6.33	AU-3000	0	0	F	Control		
	AU-3005	50bp	55x	6.27	AU-3000	0	0	M	Control		
	AU-3507	50bp	52x	6.03	AU-3500	0	0	M	Control		
	AU-3508	50bp	57x	6.51	AU-3500	0	0	F	Control		
	AU-3506	50bp	229x	26.36	AU-3500	AU-3507	AU-3508	M	Autism		
	AU-3501	50bp	209x	24.01	AU-3500	AU-3507	AU-3508	M	Autism		
	AU-4501	50bp	220x	25.34	AU-4500	AU-4503	AU-4502	M	Autism		
	AU-4502	50bp	97x	11.12	AU-4500	0	0	F	Control		
	AU-4503	50bp	88x	10.14	AU-4500	0	0	M	Control		
	AU-4504	50bp	99x	11.38	AU-4500	AU-4503	AU-4502	F	Autism		
**1 GE**	125855	91bp	42x	106.00	2-1291_5561	121530	125852	M	A	**108**	**42x**
**5 GE**	142834	91bp	37x	91.33	2-1269_5606	0	0	F	U	**89.276**	**37x**
	142836	91bp	39x	97.19	2-1269_5606	0	0	M	U		
	142833	91bp	37x	91.14	2-1269_5606	142836	142834	M	A		
	116989	91bp	35x	85.80	2-1272_5569	116985	116987	M	A		
	120099	91bp	35x	80.92	2-1276_5570	121483	120098	F	A		
**10 GE**	111582	91bp	35x	87.84	2-1239_5603	0	0	F	U	**89.153**	**36x**
	125738	91bp	37x	88.90	2-1239_5603	0	0	M	U		
	111583	91bp	37x	91.67	2-1239_5603	125738	111582	F	A		
	139150	91bp	39x	95.08	2-1244_5604	0	0	F	U		
	139151	91bp	34x	84.22	2-1244_5604	0	0	M	U		
	139152	91bp	39x	94.02	2-1244_5604	139151	139150	M	A		
	114816	91bp	36x	85.52	2-1266_5568	0	0	F	U		
	114815	91bp	34x	86.83	2-1266_5568	0	0	M	U		
	114814	91bp	35x	86.31	2-1266_5568	114815	114816	F	A		
	125733	91bp	32x	91.14	2-1295_5560	125731	125732	M	A		
**25 GE**	143817	91bp	37x	92.41	2-0704_5601	0	0	F	U	**87.4664**	**37x**
	143816	91bp	35x	84.49	2-0704_5601	0	0	M	U		

	143815	91bp	33x	79.55	2-0704_5601	143816	143817	M	A			
	89776	91bp	33x	77.57	2-1116_5214	0	0	F	U			
	89770	91bp	37x	83.71	2-1116_5214	0	0	M	U			
	89772	91bp	38x	87.70	2-1116_5214	89770	89776	M	A			
	99414	91bp	33x	83.75	2-1182_5564	0	0	F	U			
	119185	91bp	36x	89.39	2-1182_5564	0	0	M	U			
	99415	91bp	36x	86.96	2-1182_5564	119185	99414	M	A			
	129932	91bp	33x	86.07	2-1186_5565	0	0	F	U			
	129933	91bp	42x	102.78	2-1186_5565	0	0	M	U			
	129934	91bp	40x	98.66	2-1186_5565	129933	129932	M	A			
	99411	91bp	36x	91.22	2-1189_5257	0	0	F	U			
	99410	91bp	35x	82.97	2-1189_5257	0	0	M	U			
	99408	91bp	32x	71.93	2-1189_5257	99410	99411	M	A			
	124280	91bp	34x	86.30	2-1303_5574	0	0	F	U			
	129560	91bp	34x	87.39	2-1303_5574	0	0	M	U			
	124278	91bp	34x	88.31	2-1303_5574	129560	124280	M	A			
	126046	91bp	32x	75.31	2-1305_5576	0	0	F	U			
	126040	91bp	32x	87.56	2-1305_5576	0	0	M	U			
	126047	91bp	35x	87.13	2-1305_5576	126040	126046	M	A			
	128174	91bp	40x	96.75	2-1314_5577	0	0	F	U			
	128175	91bp	39x	98.27	2-1314_5577	0	0	M	U			
	128176	91bp	39x	96.75	2-1314_5577	128175	128174	M	A			
	141793	91bp	35x	83.73	2-1325_5610	141787	141784	M	A			
