# Supplementary material for: Scalable and cost-effective NGS genotyping in the cloud
Source: BMC Med Genomics. 2015 Oct 15;8:64. doi: 10.1186/s12920-015-0134-9 (PMC4608296; doi:10.1186/s12920-015-0134-9)
Supplement: Additional file 3: Table S2. — FastQC quality control table. (PDF 302 kb) [file 12920_2015_134_MOESM3_ESM.pdf]

Table S2 FastQC quality control table

	DePristo	3 Genomes	5 Genomes
NA12878	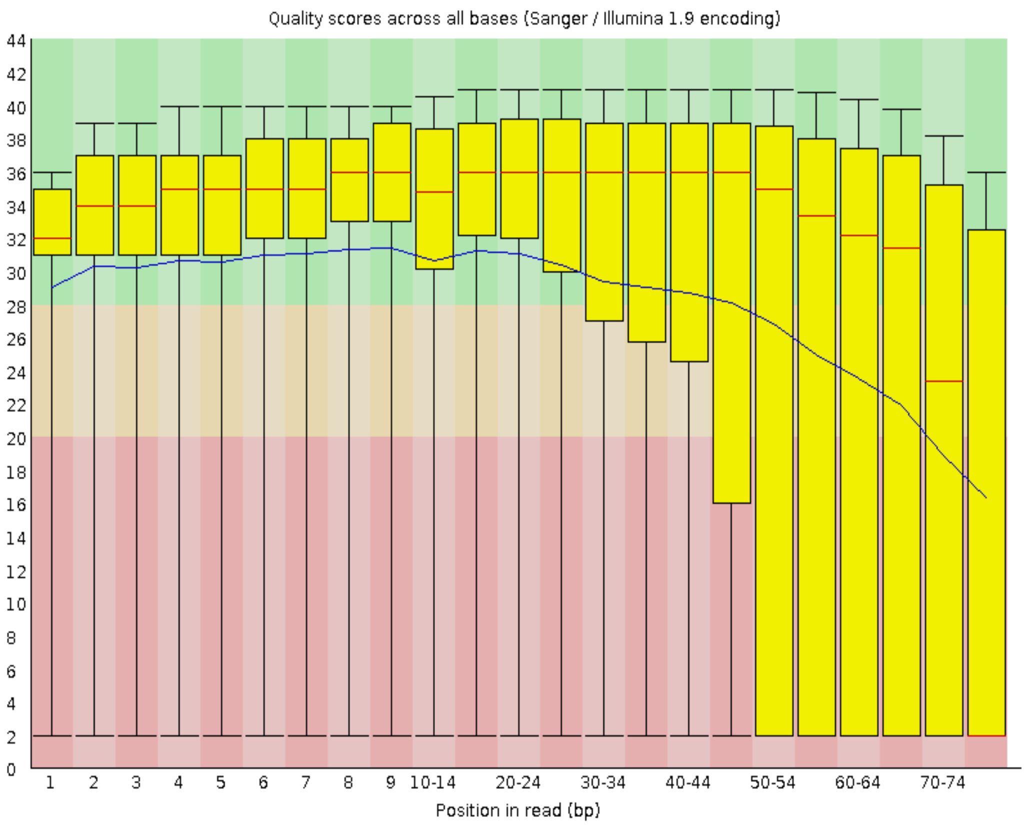	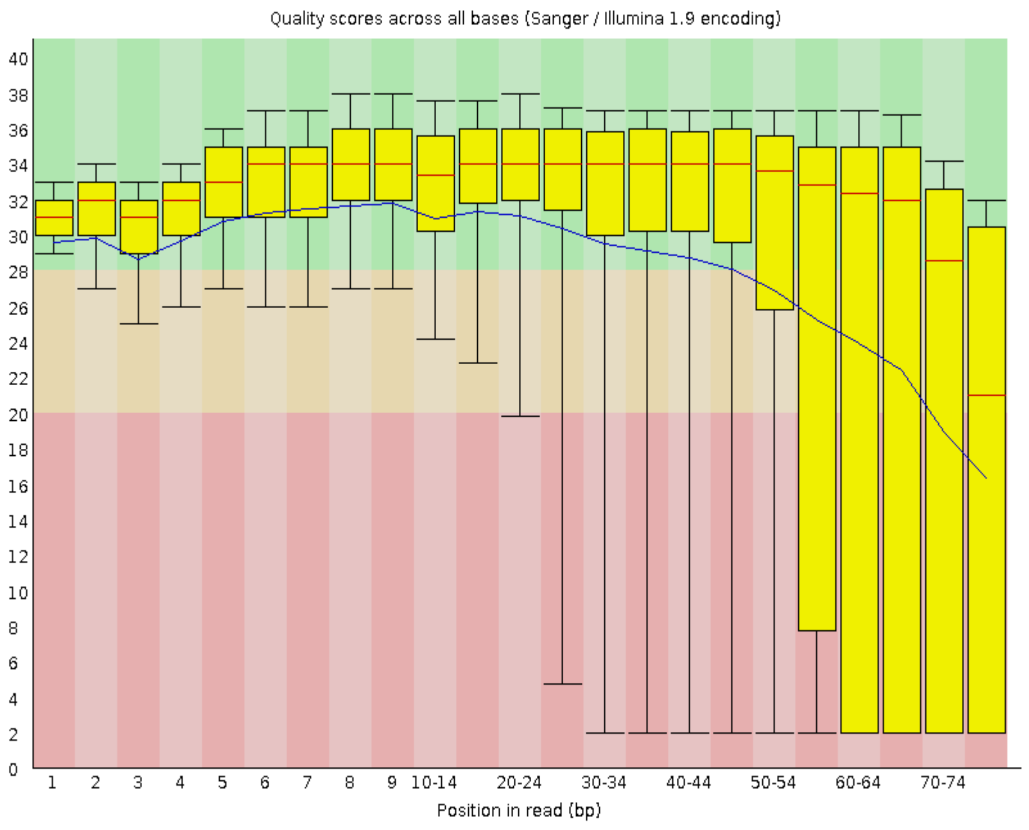	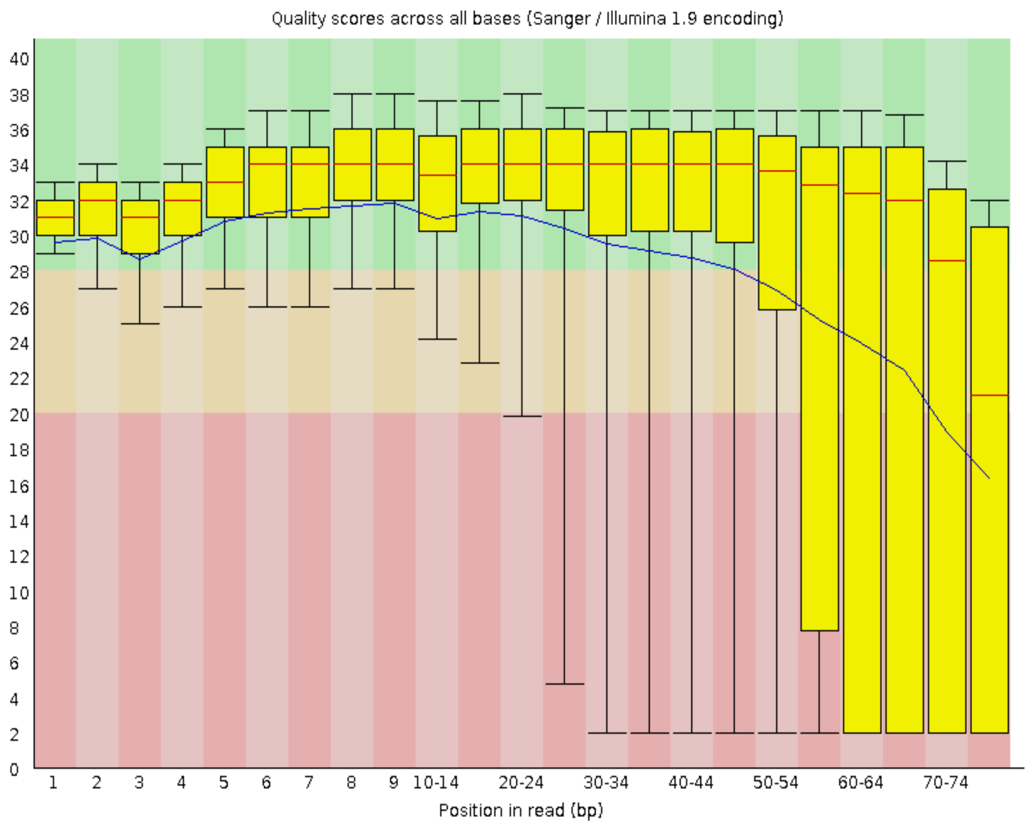
NA12891	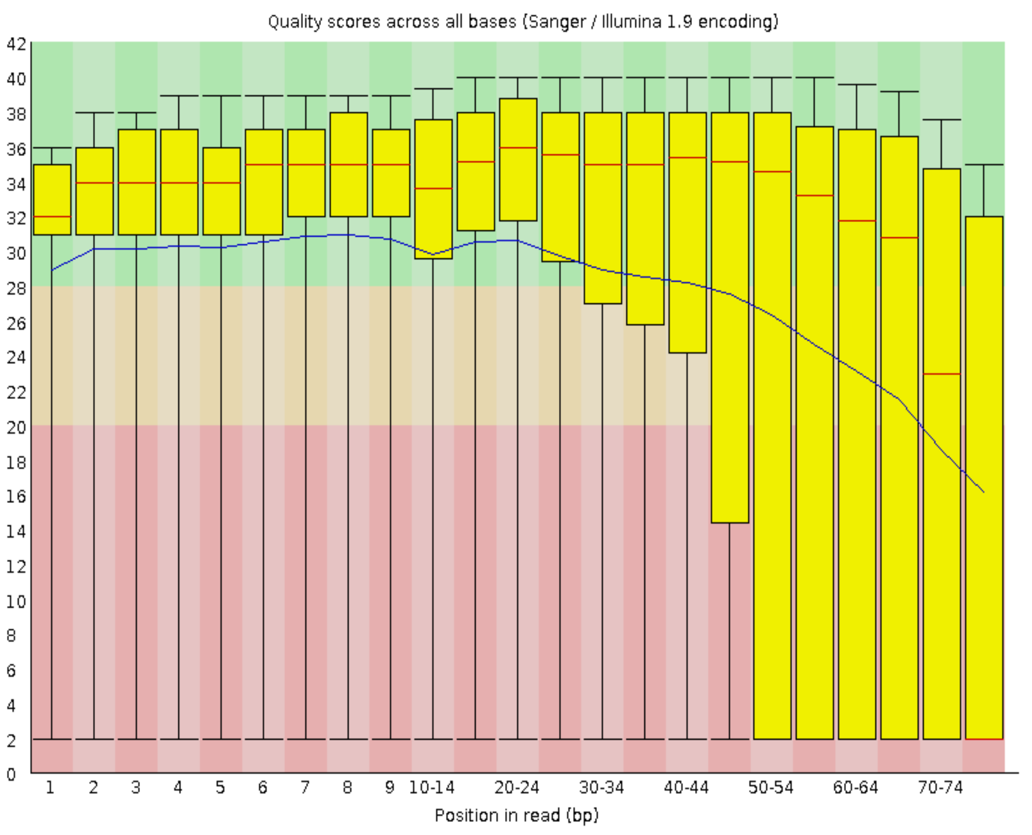	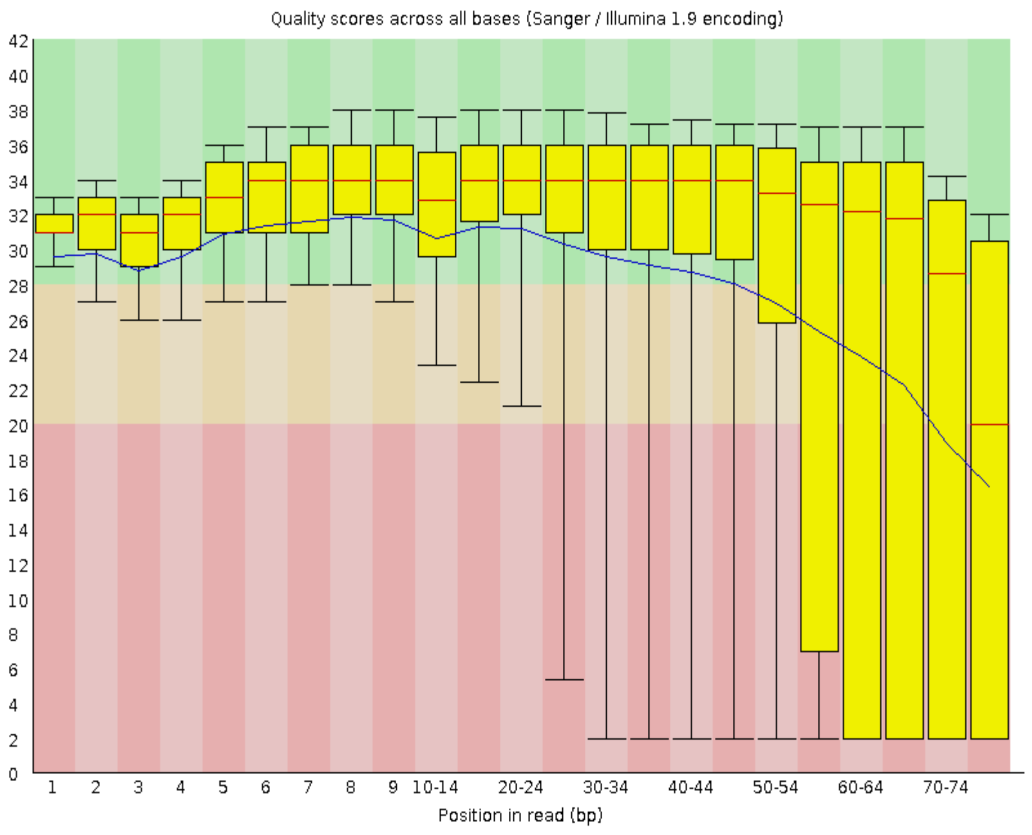	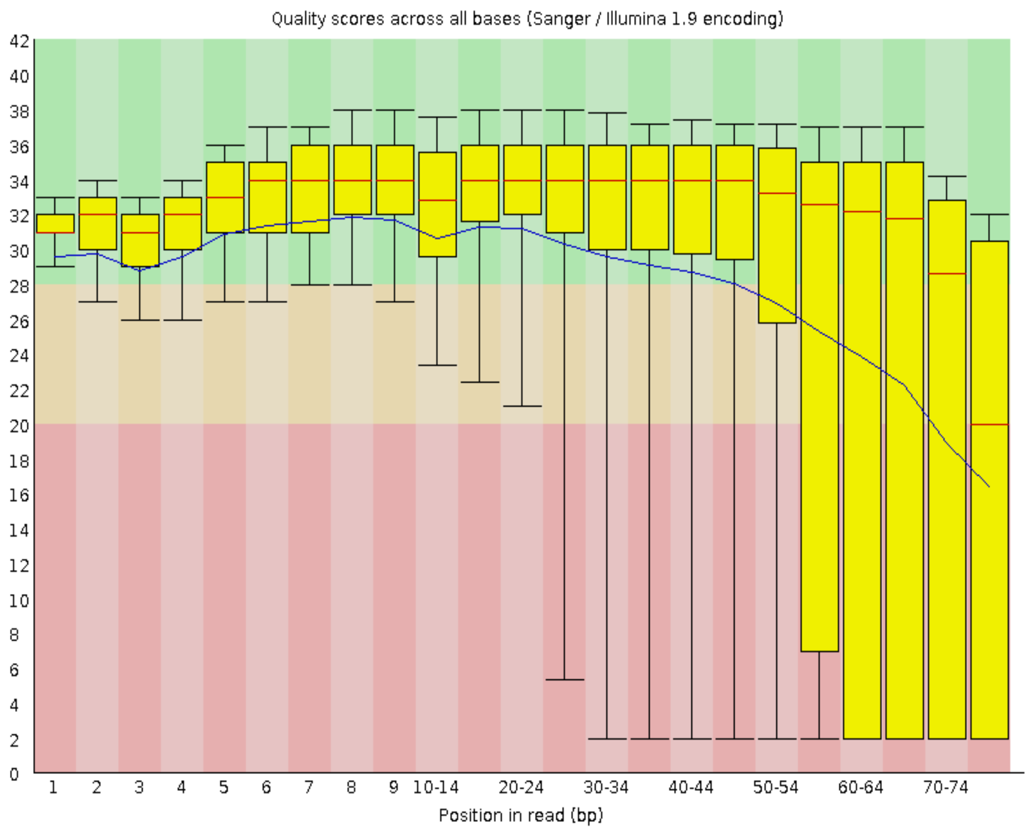
NA12892	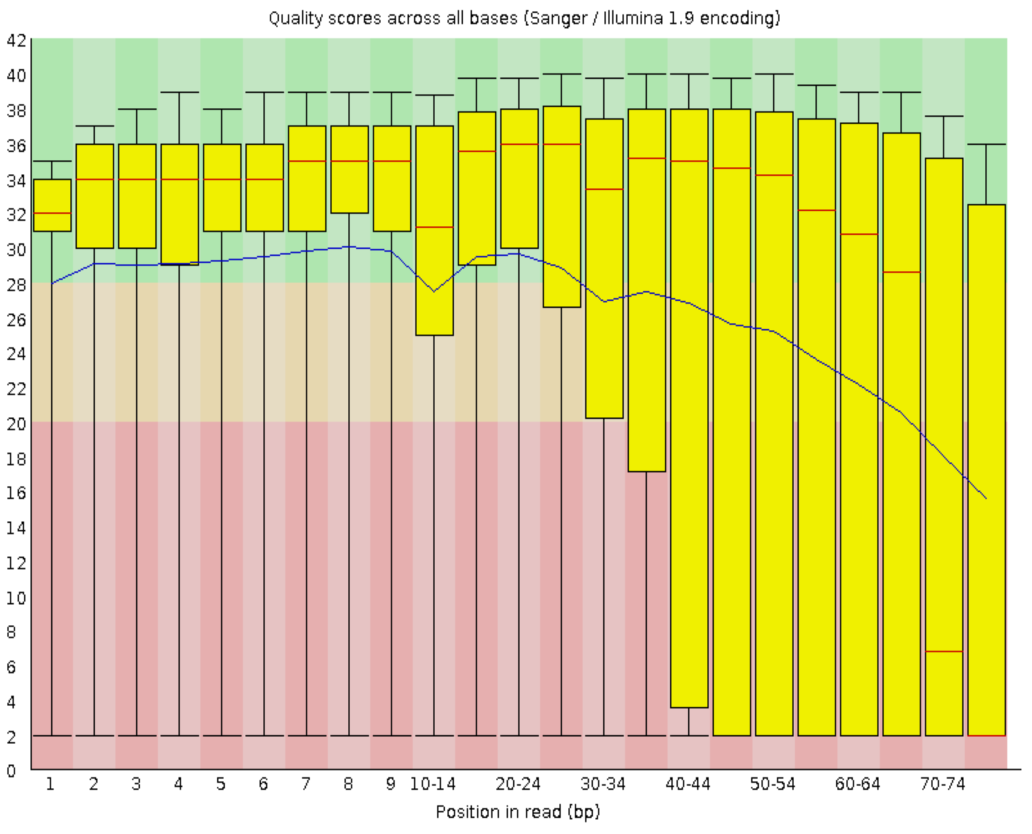	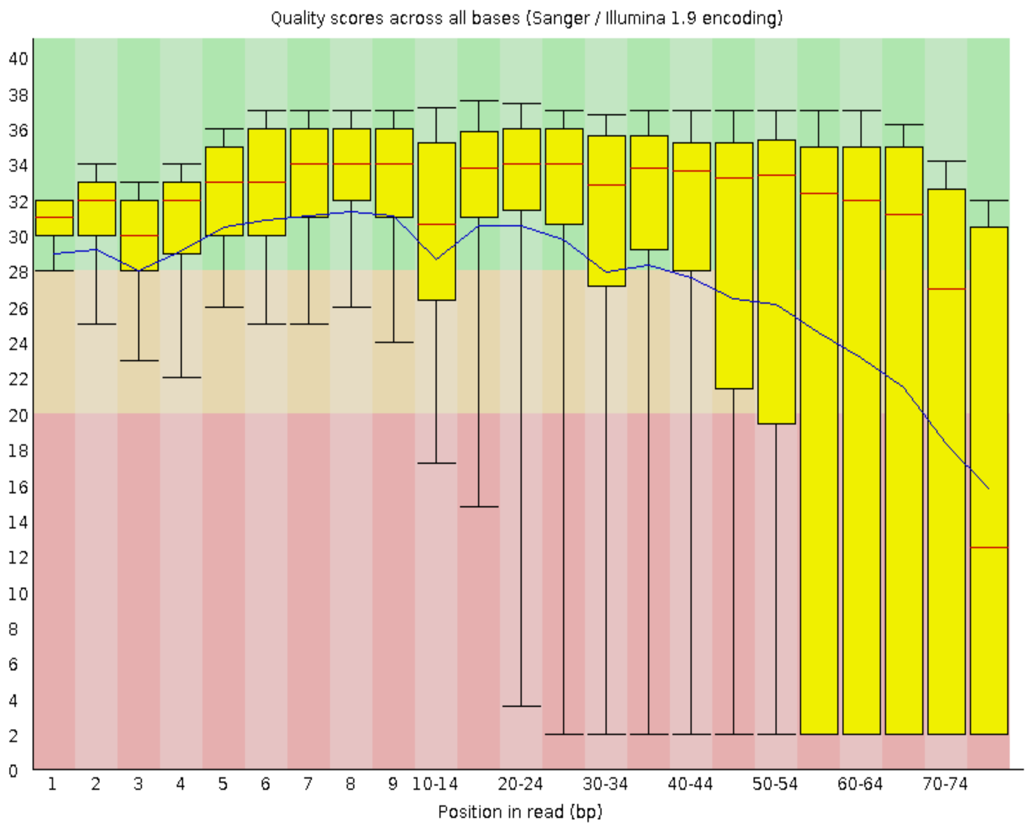	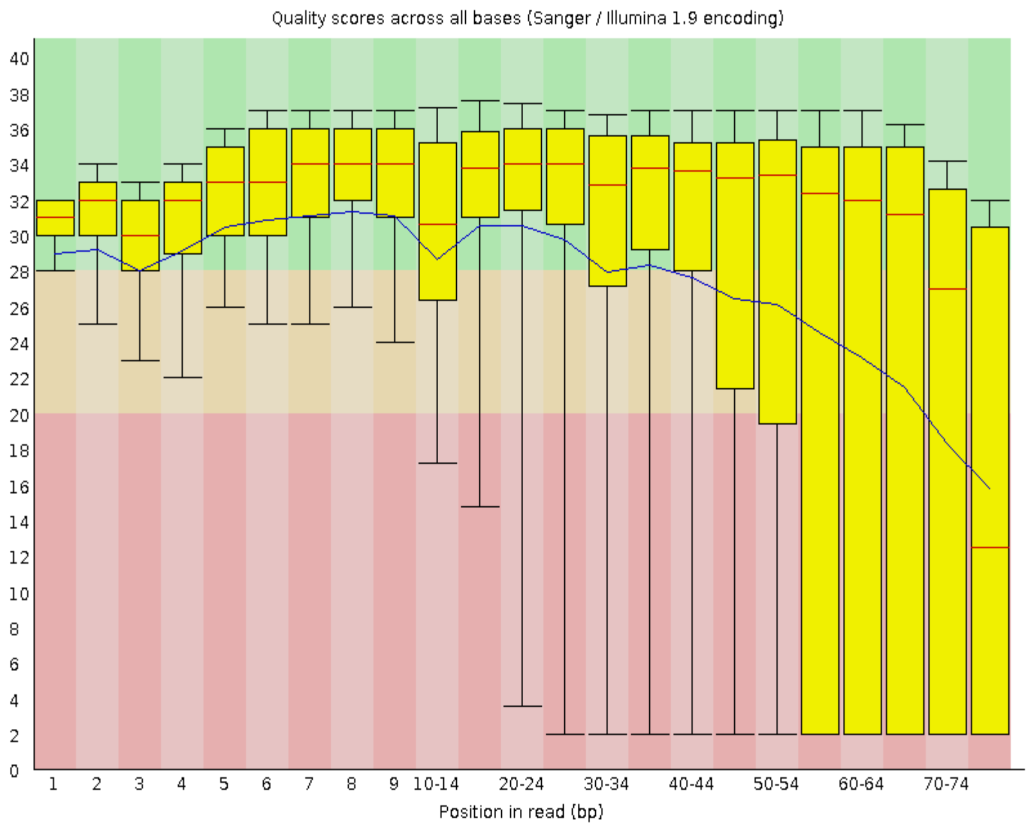
