# Supplementary material for: Scalable and cost-effective NGS genotyping in the cloud
Source: BMC Med Genomics. 2015 Oct 15;8:64. doi: 10.1186/s12920-015-0134-9 (PMC4608296; doi:10.1186/s12920-015-0134-9)
Supplement: Additional file 4: Table S3. — Comparison with existing implementations of the GATK best practices, features. (PDF 19 kb) [file 12920_2015_134_MOESM4_ESM.pdf]

Table S3 Comparison with existing implementations of the GATK best practices, features.

	Alignment	Variant-Calling	Annotation	AWS/EC2 cloud tested	Google Cloud	Joint Variant Calling Capabilities	Configured for GATK 3
**GenomeKey + COSMOS**	BWA	GATK HaplotypeCaller	ANNOVAR	Yes	Yes	Yes	Yes
**STORMseq**	BWA	GATKlite Haplotype Caller	VEP	Yes	No	Yes	No
**Rainbow**	Bowtie	SOAPsnp	None	Yes	No	No	No
**Mercury**	BWA	AtlasSNP + AtlasIndel	Cassandra	No	No	No	No
**HugeSEQ**	BWA	GATK Unified Genotyper+ Samtools	None	Yes	No	No	No
