# Supplementary material for: Scalable and cost-effective NGS genotyping in the cloud
Source: BMC Med Genomics. 2015 Oct 15;8:64. doi: 10.1186/s12920-015-0134-9 (PMC4608296; doi:10.1186/s12920-015-0134-9)
Supplement: Additional file 6: Table S4. — Runs summary with detailed cluster configuration, mean size, coverage, splitting strategy (chr: chromosome, RG: read-group), total runtime, total cost, average runtime and average cost. (PDF 61 kb) [file 12920_2015_134_MOESM6_ESM.pdf]

Table S4 Runs summary with detailed cluster configuration, mean size, coverage, splitting strategy (chr: chromosome, RG: read-group), total runtime, total cost, average runtime and average cost.

Run	Cluster configuration		Batch Size	Mean Size (GB)	Mean Coverage	Split Type	Total Runtime	Total Cost ($)	Average Runtime	Average Cost
	GlusterFS nodes (On-demand)	Worker nodes (Spot)								
1 Ex	1	20	1	**26.45**	**150x**	chr + RG	2:16:00	9.97	2:16:00	23.10
1 Ex	1	20	1	**26.45**	**150x**	chr + RG	2:16:00	23.10	2:16:00	23.10
1 Ex	1	20	1	**26.45**	**150x**	chr	2:15:00	23.10	2:15:00	23.10
1 Ex	4	17	1	**26.45**	**150x**	chr	2:06:00	23.37	2:06:00	23.37
1 Ex	4	17	1	**26.45**	**150x**	chr	2:00:52	23.37	2:00:52	23.37
1 Ex	4	17	1	**26.45**	**150x**	chr	2:01:49	23.37	2:01:49	23.37
1 Ex	4	17	1	**26.45**	**150x**	chr	2:01:41	23.37	2:01:41	23.37
1 Ex	4	17	1	**26.45**	**150x**	chr	2:05:20	23.37	2:05:20	23.37
3 Ex	1	20	3	**27.27**	**150x**	chr + RG	5:15:00	46.20	1:45:00	15.40
3 Ex	4	17	3	**27.27**	**150x**	chr	2:38:49	42.57	0:52:56	14.19
3 Ex	4	17	3	**27.27**	**150x**	chr	2:33:56	42.57	0:51:19	14.19
3 Ex	4	17	3	**27.27**	**150x**	chr	2:31:10	42.57	0:50:23	14.19
3 Ex	4	17	3	**27.27**	**150x**	chr	2:40:00	42.57	0:53:20	14.19
3 Ex	4	17	3	**27.27**	**150x**	chr	2:47:00	42.57	0:55:40	14.19
5 Ex	1	20	5	**24.68**	**162x**	chr + RG	9:06:00	77.00	1:49:12	15.40
5 Ex	4	17	5	**24.68**	**162x**	chr	3:26:50	56.76	0:41:22	11.35
5 Ex	4	17	5	**24.68**	**162x**	chr	3:25:01	56.76	0:41:00	11.35
5 Ex	4	17	5	**24.68**	**162x**	chr	3:06:43	56.76	0:37:21	11.35
5 Ex	4	17	5	**24.68**	**162x**	chr	3:33:46	56.76	0:42:45	11.35
5 Ex	4	17	5	**24.68**	**162x**	chr	3:37:51	56.76	0:43:34	11.35
10 Ex	1	20	10	**18.75**	**137x**	chr + RG	16:12:20	149.00	1:37:14	14.90
10 Ex	4	17	10	**18.75**	**137x**	chr	3:40:35	56.76	0:22:03	5.67
10 Ex	4	17	10	**18.75**	**137x**	chr	4:09:32	70.95	0:24:57	7.09
10 Ex	4	17	10	**18.75**	**137x**	chr	4:03:48	70.95	0:24:23	7.09
10 Ex	4	17	10	**18.75**	**137x**	chr	4:25:44	70.95	0:26:34	7.09
10 Ex	4	17	10	**18.75**	**137x**	chr	4:55:41	70.95	0:29:34	7.09

25 Ex	2	19	25	**17.83**	**145x**	chr	20:07:00	208.53	0:48:17	8.34
25 Ex	4	17	25	**17.83**	**145x**	chr	11:32:43	170.28	0:27:43	6.81
50 Ex	2	19	50	**17.01**	**143x**	chr	29:56:00	296.19	0:35:55	5.92
50 Ex	4	17	50	**17.01**	**143x**	chr	18:19:00	269.61	0:21:59	5.39
1 Ge	1	20	1	**108**	**42x**	chr + RG	13:52:48	109.20	13:52:48	109.20
1 Ge (Platinum)	1	20	1	**114**	**50x**	chr + RG	19:46:00	154.00	19:46:00	154.00
3 Ge (Platinum)	1	20	3	**113.62**	**50x**	chr + RG	35:10:09	280.80	11:43:23	93.60
5 Ge	2	19	5	**89.276**	**37x**	chr + RG	31:31:07	377.34	6:18:13	75.46
10 Ge	4	17	10	**89.15**	**36x**	chr + RG	37:48:39	532.76	3:46:52	53.27
25 Ge	4	17	25	**87.47**	**37x**	chr + RG	121:56:07	1213.39	4:52:39	48.53
