# Supplementary material for: Scalable and cost-effective NGS genotyping in the cloud
Source: BMC Med Genomics. 2015 Oct 15;8:64. doi: 10.1186/s12920-015-0134-9 (PMC4608296; doi:10.1186/s12920-015-0134-9)
Supplement: Additional file 7: Table S5 — Comparison with previous benchmarks for specific 1000 g exomes/genomes. (PDF 26 kb) [file 12920_2015_134_MOESM7_ESM.pdf]

Table S5 Comparison with previous benchmarks for specific 1000g exomes/genomes.

Published runtimes	Dataset/Type	Coverage	Time	Aligner	Variant Caller	Hardware Specs	Cost
Genomekey + COSMOS	25x Personal Genomes	37X	122 Hours	BWA	GATK HaplotypeCaller	20x AWS cc2.8xlarge workers	$48.53 per genome, $1.2K total
Churchill	1088x 1000 Genome Project	5X	168 Hours	BWA	GATK Unified Genotyper	400x AWS cc2.8xlarge workers	Not Reported
STORMseq Genome	1x Personal (Unpublished) Genome	38X	176 Hours	BWA	GATK lite	Not Reported	$32.76
STORMseq Exome	1x Personal (Unpublished) Exome	45X	10 Hours	BWA	GATK lite	Not Reported	$1.90
Crossbow	1x Yan-Huang Genome	38X	4 Hours	Bowtie	SOAPsnp	20x AWS c1.xlarge workers	$71.40
Rainbow	44x Personal Genomes	30X - 60X	2 Weeks Total	Bowtie	SOAPsnp	40x AWS c1.xlarge workers per Genome	<120$ per genome, 5.8K total
Mercury	1x NA12878 Exome	150X	1 Day	BWA	AtlasSNP/ AtlasIndel	8-cores 32Gb RAM	Not Reported
HugeSEQ	Reads from B&S Seq Archive	48X	38 Hours	BWA	GATK+Samtools+Additional CNV calling tools	48 cores, 12Gb RAM	Not Reported
SIMPLEX	10x Kabuki Syndrome Exomes	40X	25 Hours per exome	BWA	Custom Made	128 cores, 1Tb RAM	Not Reported
Atlas2	1x NA19093 Exome	~30X	8 Hours	None	AtlasSNP2/AtlasIndel	1x AWS m1.large	$5.00
